# Supplementary material for: Diurnal cloud cycle biases in climate models
Source: Nat Commun. 2017 Dec 22;8:2269. doi: 10.1038/s41467-017-02369-4 (PMC5741665; doi:10.1038/s41467-017-02369-4)
Supplement: Supplementary file 1 — Supplementary Information [file 41467_2017_2369_MOESM1_ESM.pdf]

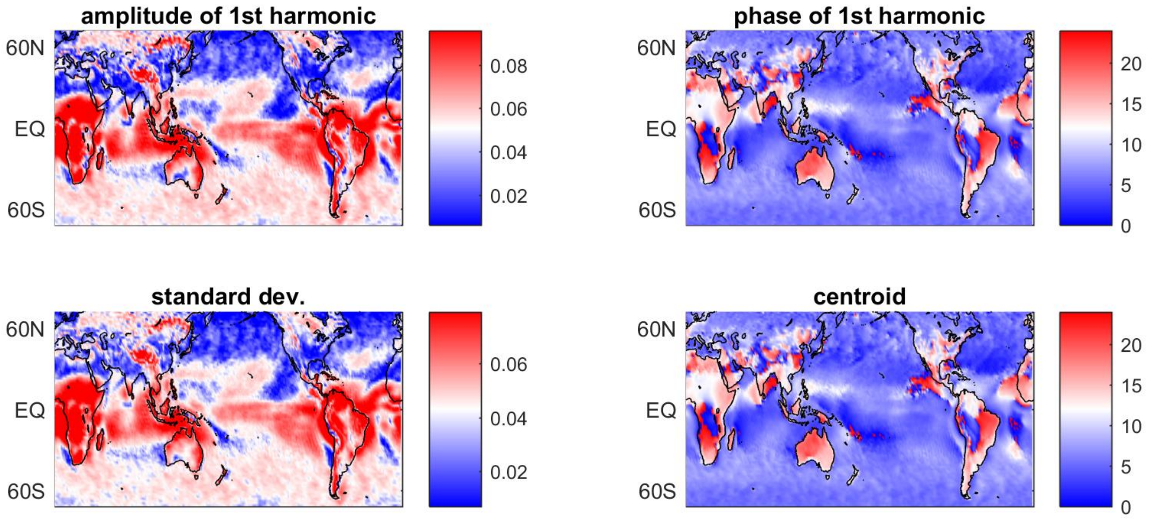

Supplementary Figure 1 DCC indexes and amplitude/phase of the first harmonic in winter (DJF) from CNRM-CM5 model.

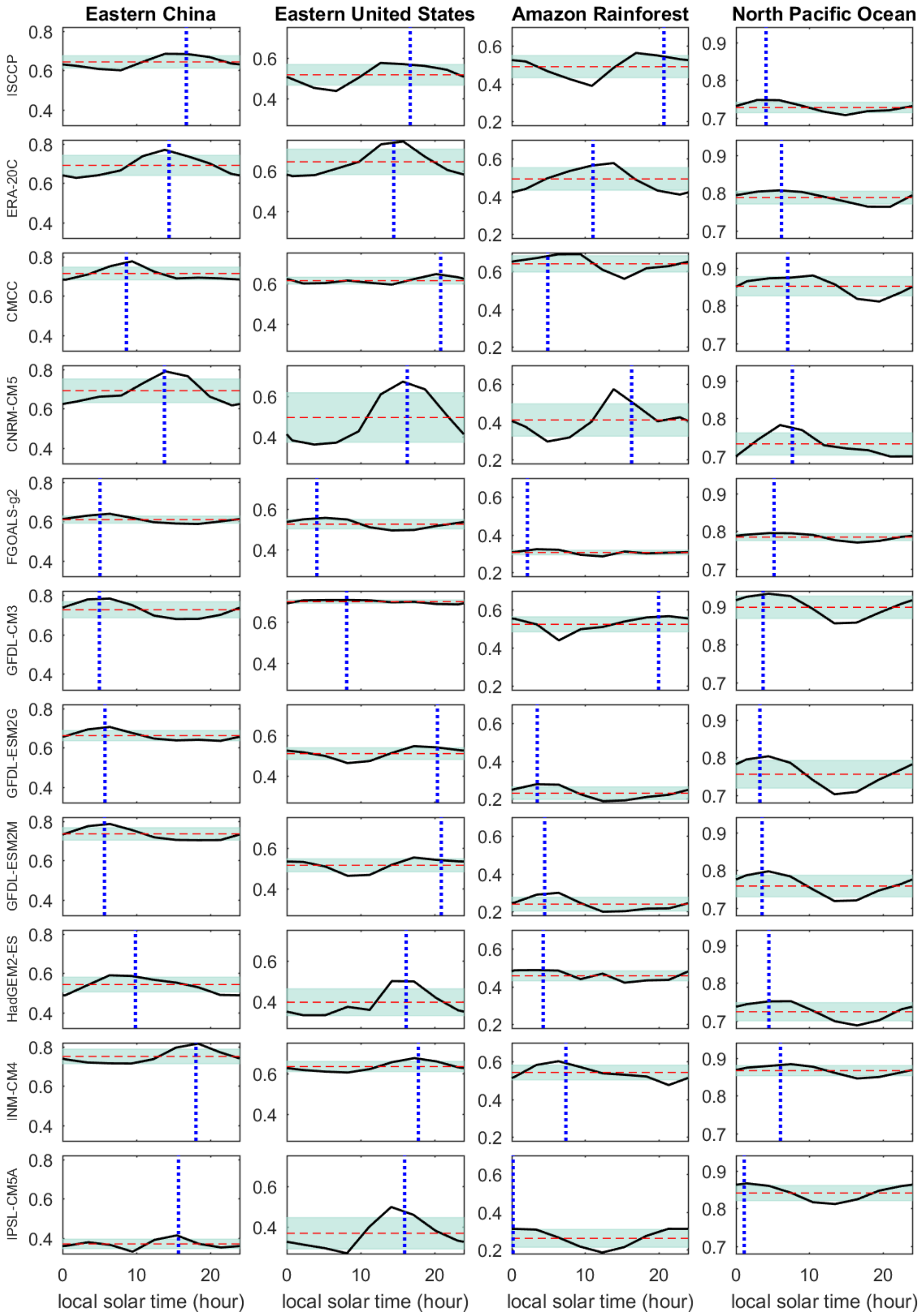

Supplementary Figure 2 Examples of diurnal cycle of average cloud coverage in summer (June, July, and August) during 1986-2005 from ISCCP records (1st row), ERA-20C reanalysis (2nd row), and GCMs historical experiment (3rd-11th row), near four locations (1st-4th column): Guangde, China (30.7N, 119.2E), Durham, USA (36N,79.9W), Codajás, Brazil (3.5S, 62.2W), and North Pacific Ocean (36S, 180E). The vertical dot lines and horizontal dash lines show the centroid and mean of the diurnal cycle climatology; the shaded blue areas indicate plus and minus one standard deviation.

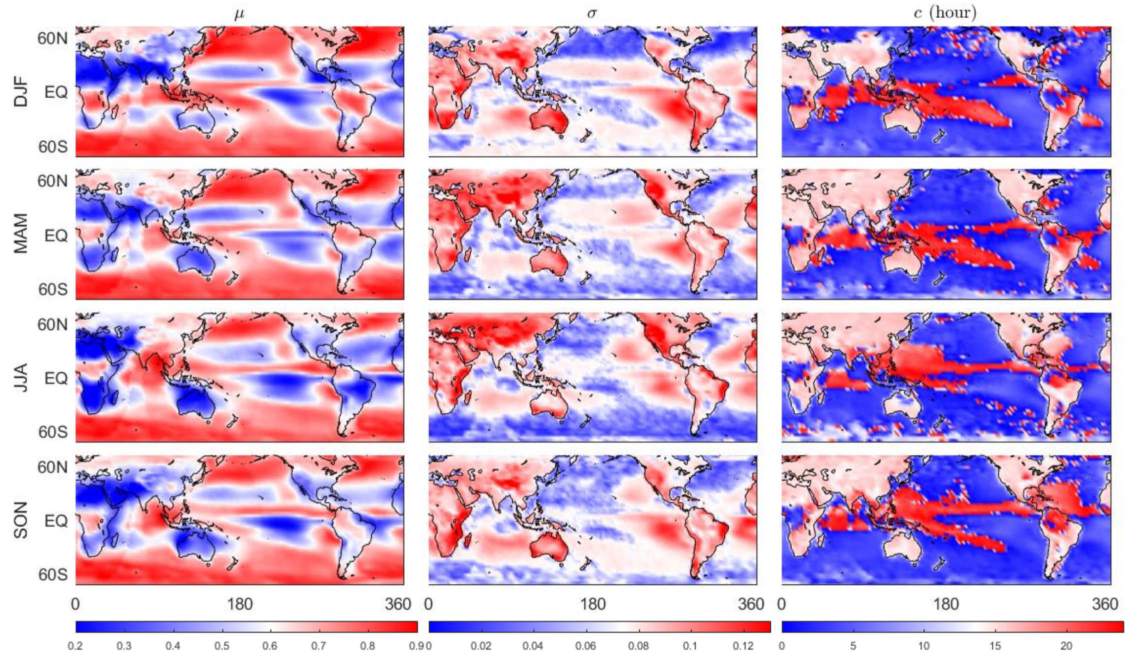

Supplementary Figure 3 Indexes of diurnal cycle of clouds from ISCCP cloud climatology during 1986-2005. Left to right columns show the mean, standard deviation, and centroid of the diurnal cycle of clouds; Top to bottom rows are in winter, spring, summer, and fall seasons. Note that the change of *c* from 24 hr to 0 hr should be interpreted as a smooth transition (e.g. in the tropical Pacific).

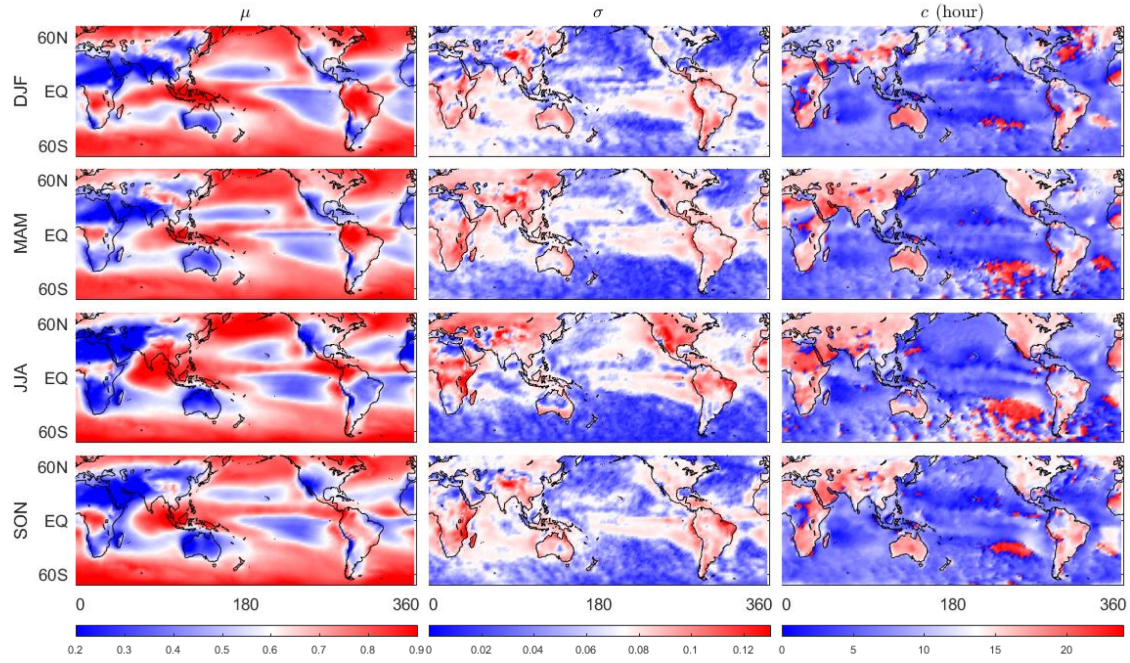

Supplementary Figure 4 As in Supplementary Figure 3 but for ERA-20C cloud climatology.

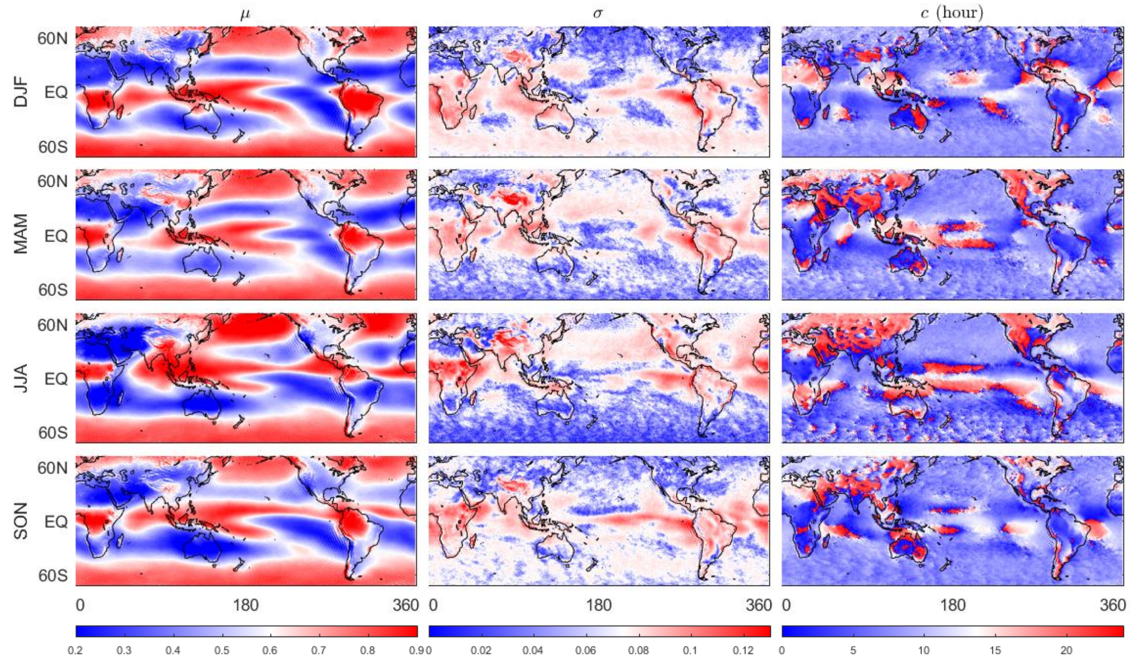

Supplementary Figure 5 As in Supplementary Figure 3 but for CMCC-CM cloud climatology.

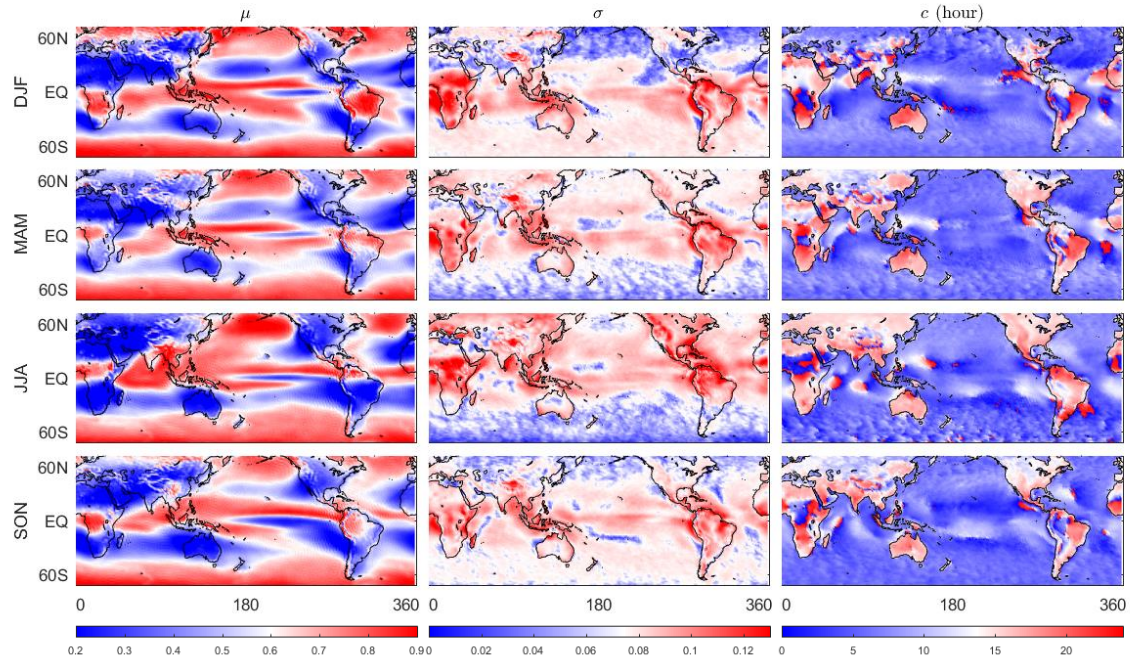

Supplementary Figure 6 As in Supplementary Figure 3 but for CNRM-CM5 cloud climatology.

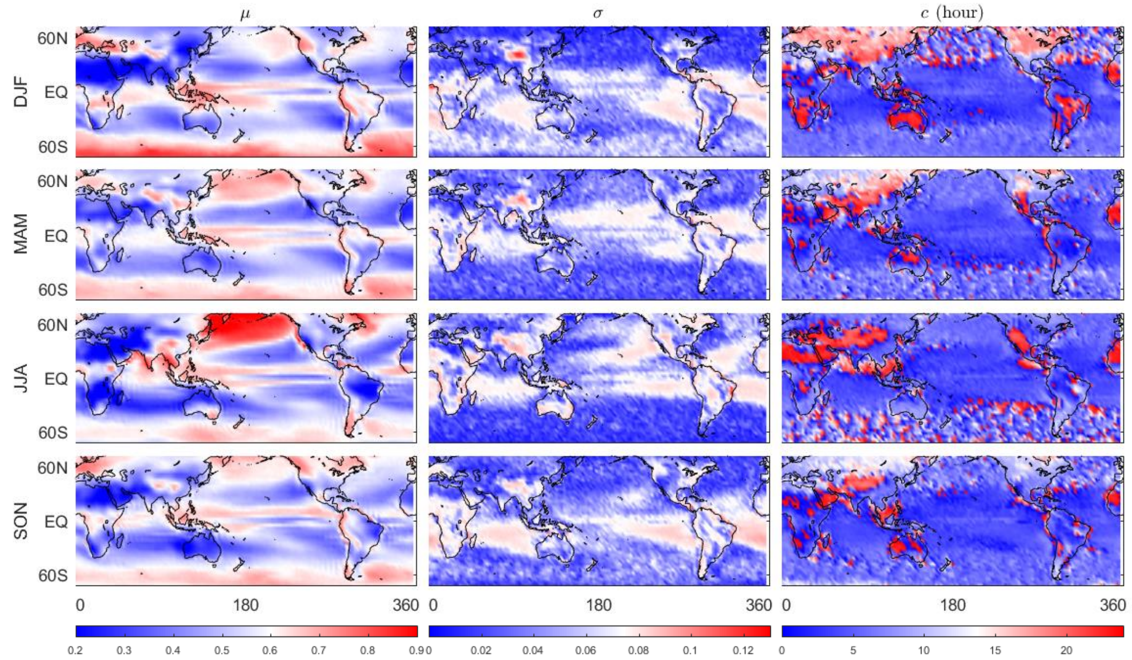

Supplementary Figure 7 As in Supplementary Figure 3 but for FGOALS-g2 cloud climatology.

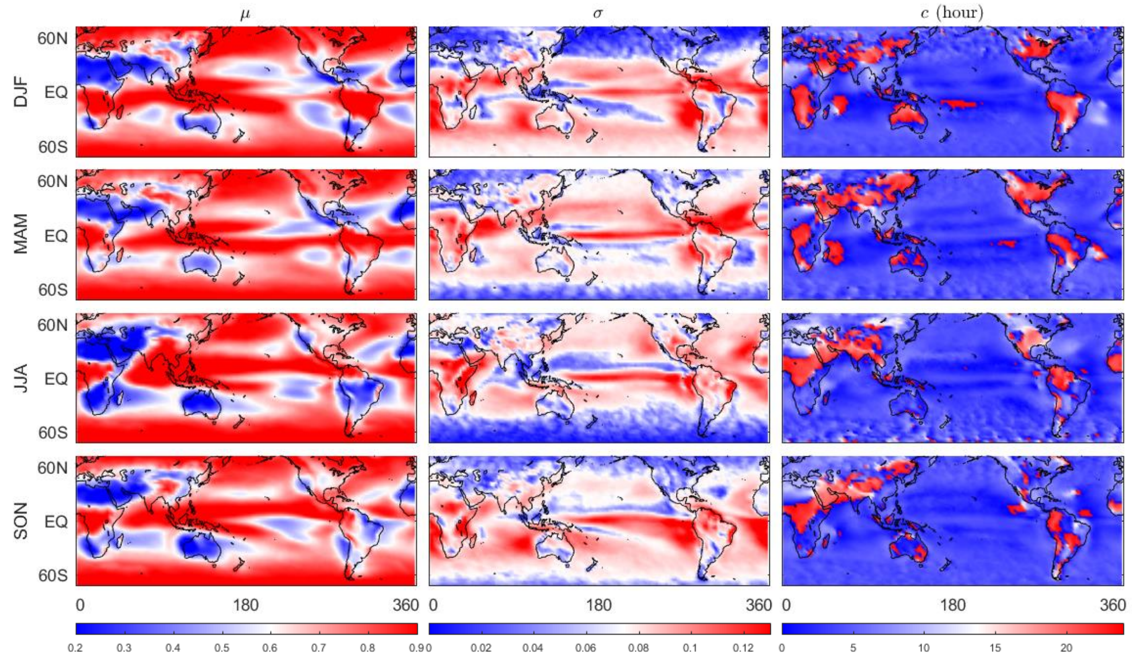

Supplementary Figure 8 As in Supplementary Figure 3 but for GFDL-CM3 cloud climatology.

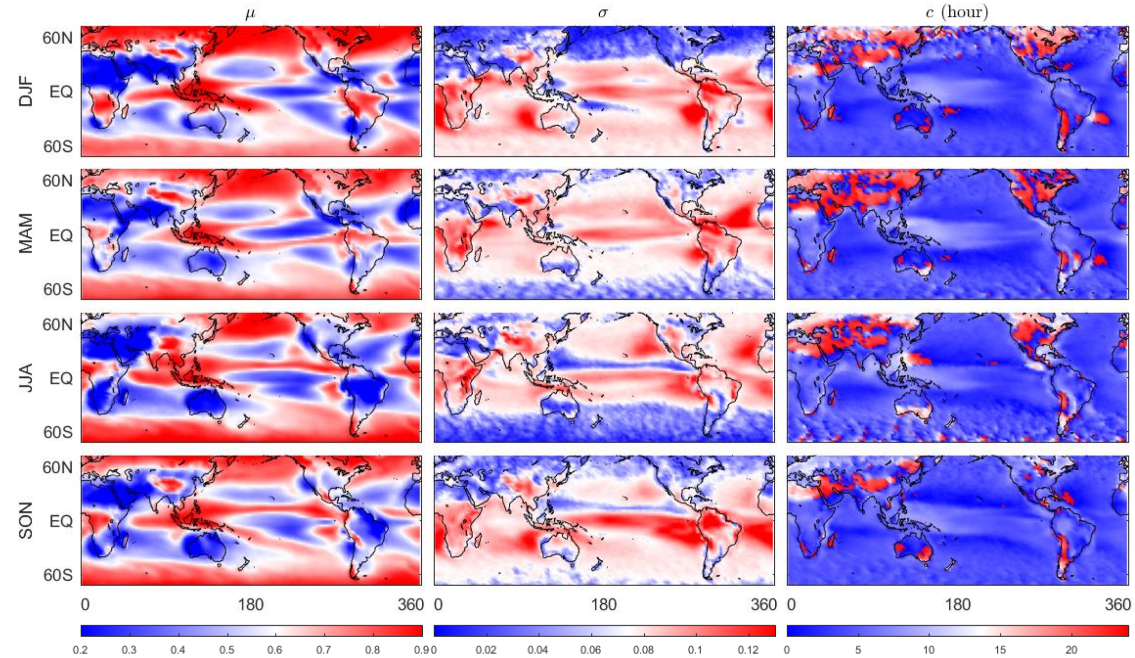

Supplementary Figure 9 As in Supplementary Figure 3 but for GFDL-ESM2G cloud climatology.

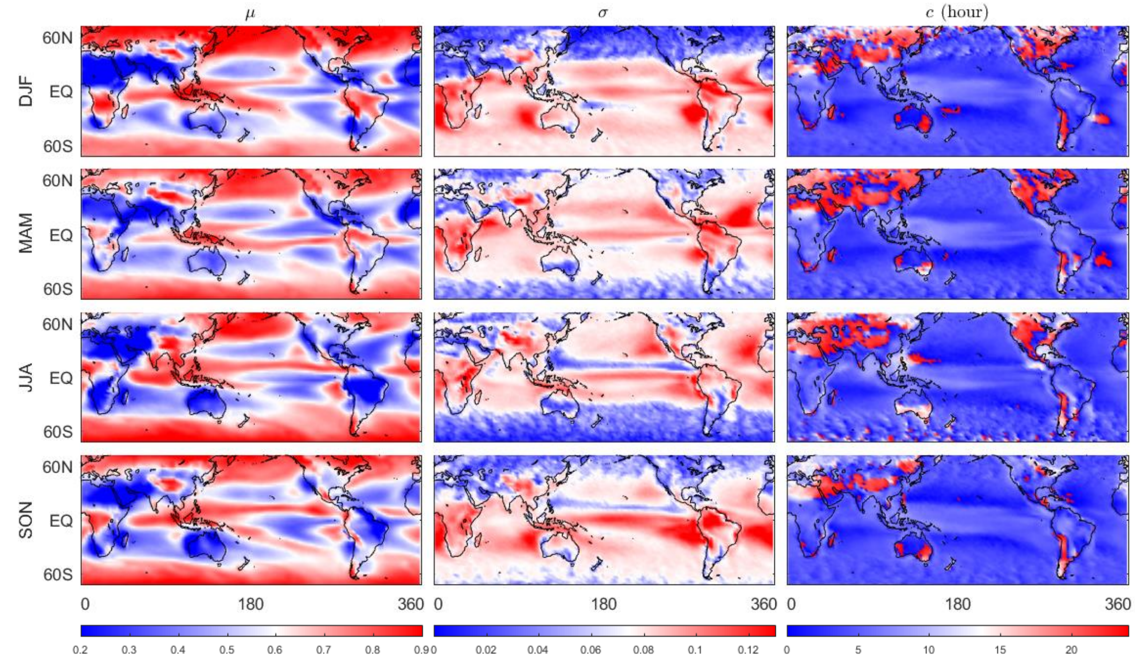

Supplementary Figure 10 As in Supplementary Figure 3 but for GFDL-ESM2M cloud climatology.

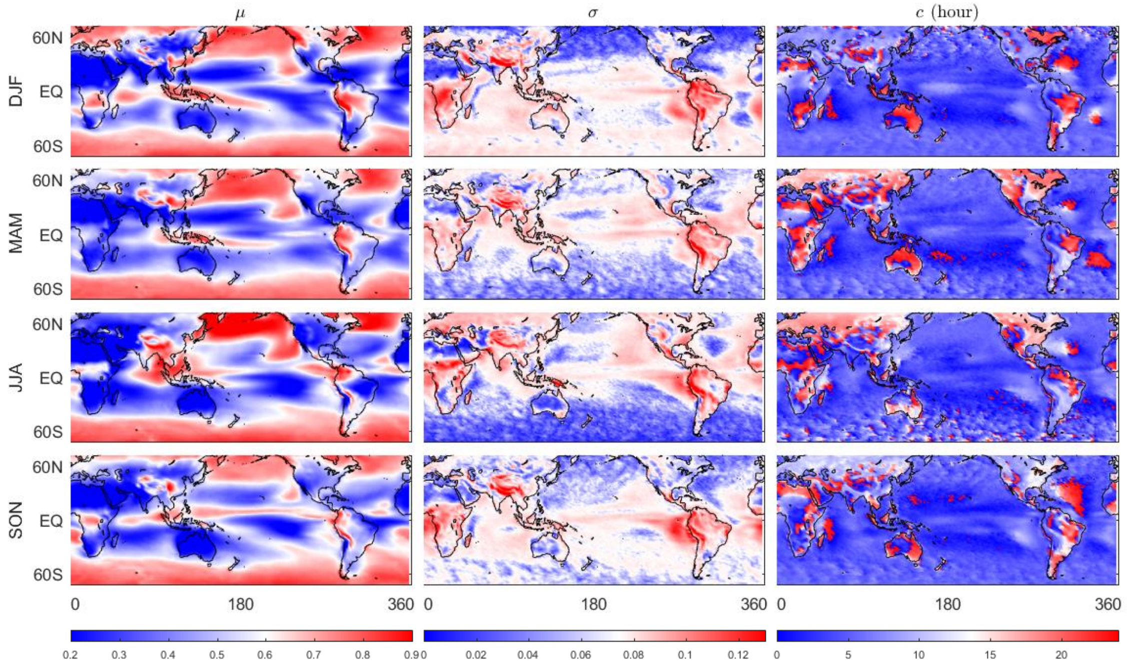

Supplementary Figure 11 As in Supplementary Figure 3 but for HadGEM2-ES cloud climatology.

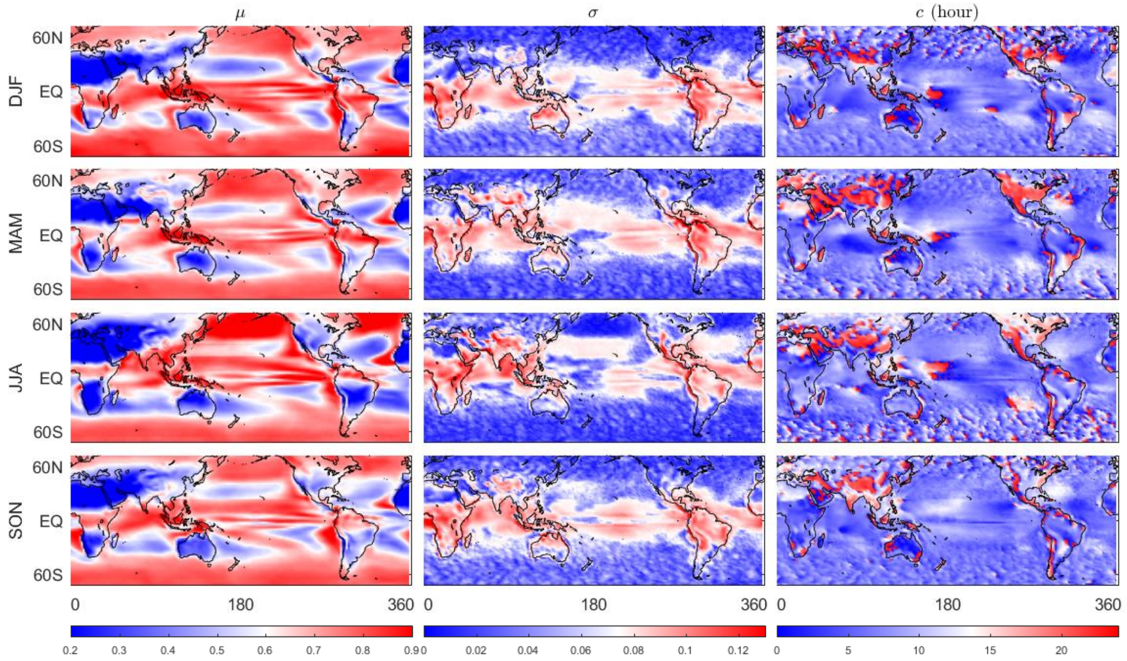

Supplementary Figure 12 As in Supplementary Figure 3 but for INM-CM4 cloud climatology.

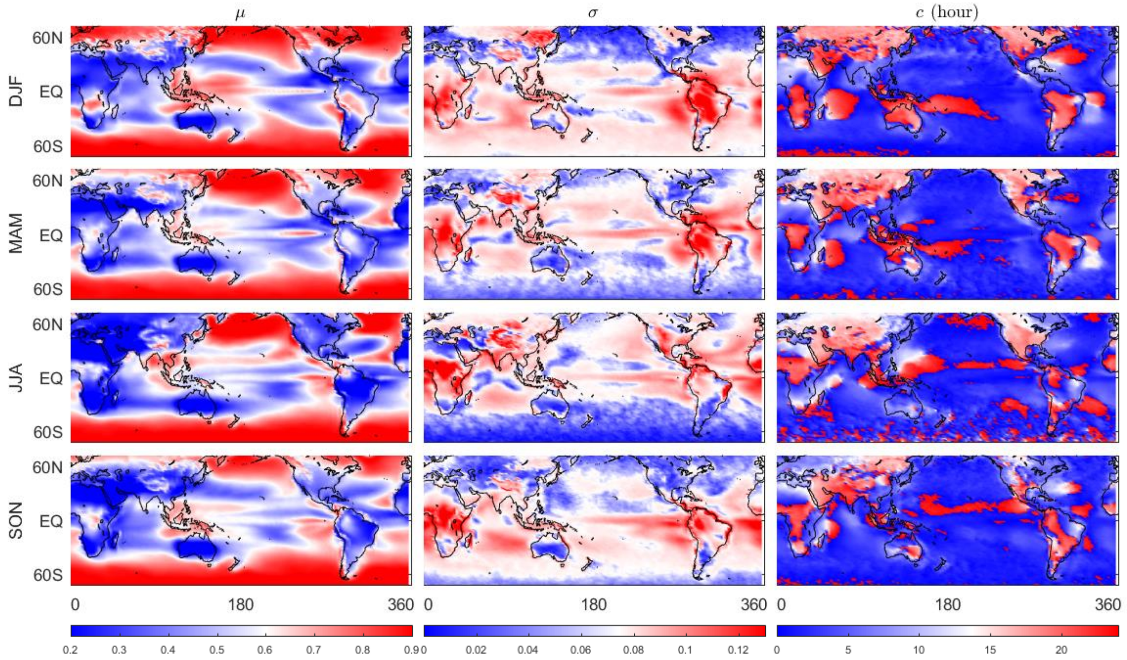

Supplementary Figure 13 As in Supplementary Figure 3 but for IPSL-CM5A-MR cloud climatology.

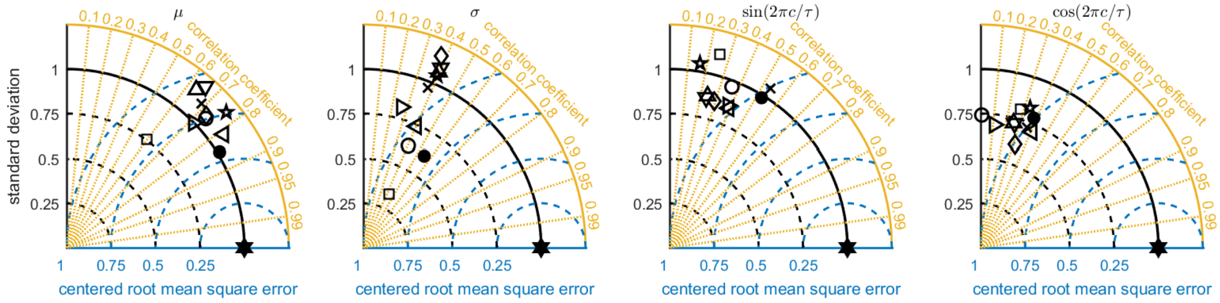

Supplementary Figure 14 | Normalized Taylor Diagram for the global spatial patterns of cloud diurnal cycle climatology in winter (December, January, and February). The circular variable centroid (c) is converted to cartesian coordinates $[\sin(2\pi/\tau)\ ,\ \cos(2\pi/\tau)]$ to produce the corresponding Taylor diagrams. Similar methods have been used for comparing wind field, which is usually decomposed into zonal and meridional components[25]. For mean (μ) and standard deviation (σ), the whole global regions with equal-area grids are used for producing the Taylor diagrams; For centroid (c), only regions with relative stronger diurnal cycles (here, we assume c_v larger than its 25th percentile) are used for producing the Taylor diagram. Detailed symbols and their corresponding data sources are listed in supplementary Table 1.

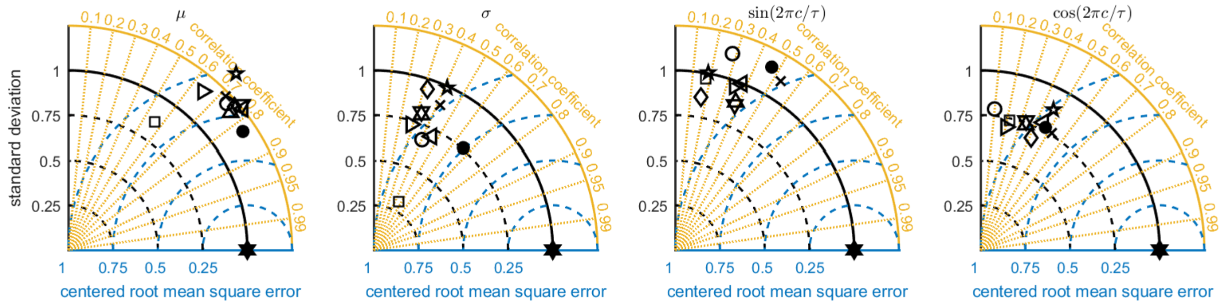

Supplementary Figure 15 As in Supplementary Figure 14, but for summer (June, July, and August).

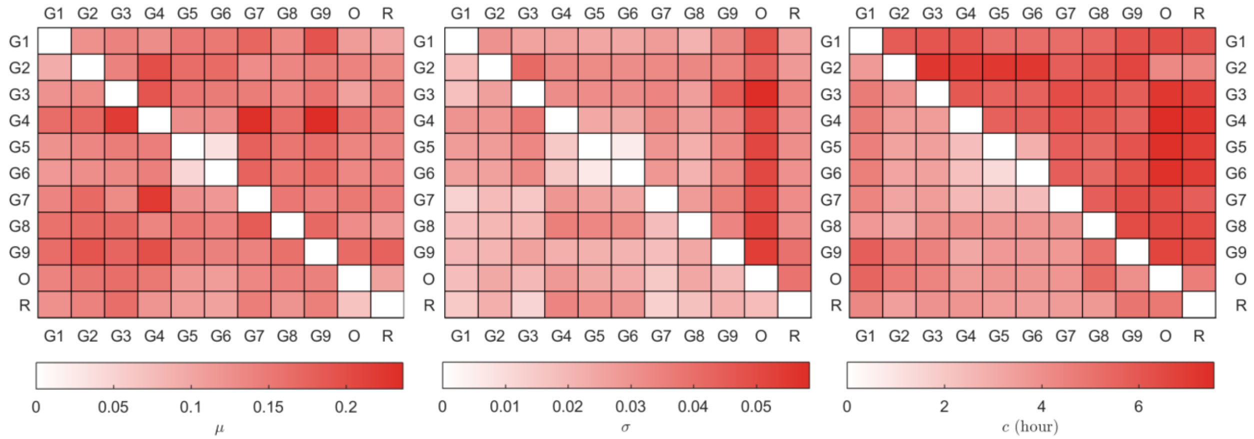

Supplementary Figure 16 Root-mean-square deviation (RMSD) of mean (μ), standard deviation (σ), and centroid (c) of diurnal cycle of cloud coverage. Each element in the color matrix is corresponding to the RMSD from two data sources marked by the abbreviations on the axes. The lower and upper triangle matrixes are for the RMSD over the ocean and land, respectively, and the diagonal matrix, RMSD in itself, is zero by definition. The abbreviations and their corresponding data source names are listed in supplementary Table 1.

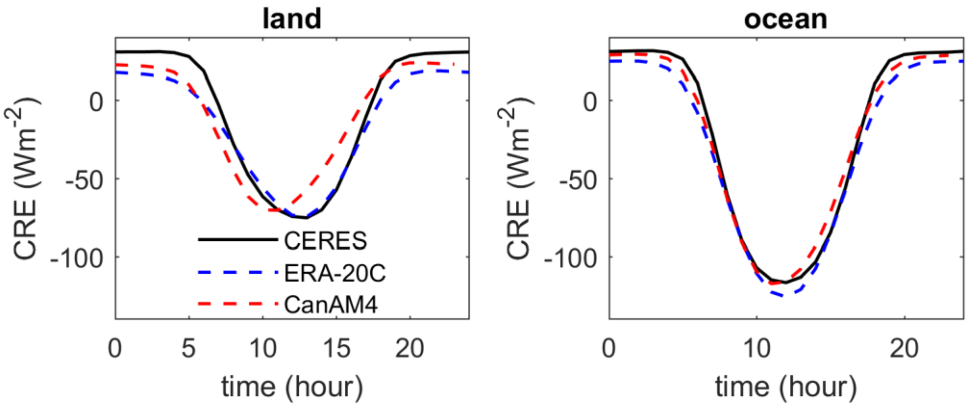

Supplementary Figure 17 Comparison of diurnal cycle of CRE climatology over the land and ocean from Clouds and the Earth's Radiant Energy System (CERES), ERA-20C, and CanAM4. In general, these diurnal cycles have similar shapes but CRE from ERA-20C and CanAM4 are smaller than CRE from CERES.

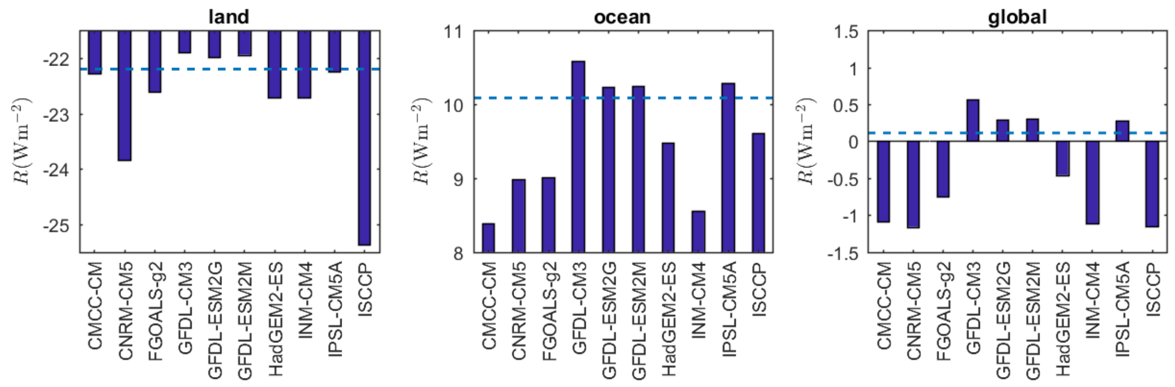

Supplementary Figure 18 As in Fig. 4 in the main text, but for baseline μ and CRE calculated from CanAM4 AMIP experiment.

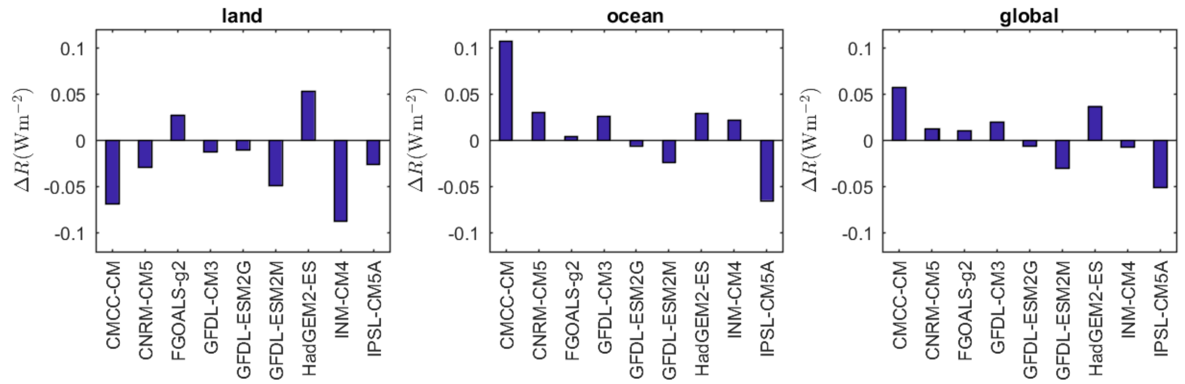

Supplementary Figure 19 As in Fig. 5 in the main text, but for baseline μ and CRE calculated from CanAM4 AMIP experiment.

Supplementary Table 1 Climate Models, satellite observations, and reanalysis data used for assessing cloud diurnal cycles

#	symbol	acronyms	model institutions and references
1	○	CMCC-CM	Euro-Mediterranean Center on Climate Change, Italy
2	×	CNRM-CM5	National Center for Meteorological Research, France
3	□	FGOALS-g2	LASG, Institute of Atmospheric Physics, Chinese Academy of Sciences, China; CESS, Tsinghua University, China
4	◇	GFDL-CM3	NOAA Geophysical Fluid Dynamics Laboratory, USA
5	▽	GFDL-ESM2G	NOAA Geophysical Fluid Dynamics Laboratory, USA
6	△	GFDL-ESM2M	NOAA Geophysical Fluid Dynamics Laboratory, USA
7	◁	HadGEM2-ES	Met Office Hadley Centre, United Kingdom
8	▷	INM-CM4	Institute for Numerical Mathematics, Russia
9	☆	IPSL-CM5A	Institute Pierre Simon Laplace, France
O	✶	ISCCP	National Aeronautics and Space Administration, USA
R	●	ERA-20C	European Centre for Medium-Range Weather Forecasts
